# The Cysteine-Rich Interdomain Region from the Highly Variable *Plasmodium falciparum* Erythrocyte Membrane Protein-1 Exhibits a Conserved Structure

**DOI:** 10.1371/journal.ppat.1000147

**Published:** 2008-09-05

**Authors:** Michael M. Klein, Apostolos G. Gittis, Hua-Poo Su, Morris O. Makobongo, Jaime M. Moore, Sanjay Singh, Louis H. Miller, David N. Garboczi

**Affiliations:** 1 Structural Biology Section, Laboratory of Immunogenetics, National Institute of Allergy and Infectious Diseases, National Institutes of Health, Rockville, Maryland, United States of America; 2 Malaria Vaccine Development Branch, National Institute of Allergy and Infectious Diseases, National Institutes of Health, Rockville, Maryland, United States of America; Weill Medical College of Cornell University, United States of America

## Abstract

*Plasmodium falciparum* malaria parasites, living in red blood cells, express proteins of the erythrocyte membrane protein-1 (PfEMP1) family on the red blood cell surface. The binding of PfEMP1 molecules to human cell surface receptors mediates the adherence of infected red blood cells to human tissues. The sequences of the 60 PfEMP1 genes in each parasite genome vary greatly from parasite to parasite, yet the variant PfEMP1 proteins maintain receptor binding. Almost all parasites isolated directly from patients bind the human CD36 receptor. Of the several kinds of highly polymorphic cysteine-rich interdomain region (CIDR) domains classified by sequence, only the CIDR1α domains bind CD36. Here we describe the CD36-binding portion of a CIDR1α domain, MC179, as a bundle of three α-helices that are connected by a loop and three additional helices. The MC179 structure, containing seven conserved cysteines and 10 conserved hydrophobic residues, predicts similar structures for the hundreds of CIDR sequences from the many genome sequences now known. Comparison of MC179 with the CIDR domains in the genome of the *P. falciparum* 3D7 strain provides insights into CIDR domain structure. The CIDR1α three-helix bundle exhibits less than 20% sequence identity with the three-helix bundles of Duffy-binding like (DBL) domains, but the two kinds of bundles are almost identical. Despite the enormous diversity of PfEMP1 sequences, the CIDR1α and DBL protein structures, taken together, predict that a PfEMP1 molecule is a polymer of three-helix bundles elaborated by a variety of connecting helices and loops. From the structures also comes the insight that DBL1α domains are approximately 100 residues larger and that CIDR1α domains are approximately 100 residues smaller than sequence alignments predict. This new understanding of PfEMP1 structure will allow the use of better-defined PfEMP1 domains for functional studies, for the design of candidate vaccines, and for understanding the molecular basis of cytoadherence.

## Author Summary

Malaria parasites express proteins of the erythrocyte membrane protein-1 family (PfEMP1) on the surfaces of the human red blood cells that they infect. These large proteins vary in sequence extensively, yet bind to host receptors to allow infected cells to adhere to host tissues. PfEMP1 proteins help parasites evade the immune system, as the 60 PfEMP1 genes are expressed one at a time. Sequence comparisons predict that PfEMP1 molecules are modular, made up of Duffy binding-like (DBL) and cysteine-rich interdomain region (CIDR) domains. Many CIDR domains bind to the human receptor CD36. We have analyzed the structure of the CD36-binding portion, known as MC179, of a CIDR domain. The MC179 protein is composed of a bundle of three helices connected by a loop and three additional helices. Based on the structure and sequence similarities, MC179 is a good model for the hundreds of known CIDR sequences. In addition, the MC179 three-helix bundle is remarkably similar to subdomain 3 of the known DBL structures. MC179 provides insight into the relatedness of both kinds of PfEMP1 domains and predicts that the large PfEMP1 molecules are polymers of three-helix bundles and their connecting polypeptides.

## Introduction

Cycles of exponential parasite growth inside infected red blood cells (iRBCs), followed by lysis, and immediate re-invasion of uninfected RBCs are the hallmarks of malaria caused by *P. falciparum*. Though seemingly hidden from the human immune system inside the iRBC, *P. falciparum* expresses a number of proteins on the iRBC surface, extensively remodeling the iRBC, and exposing parasite antigens to the immune system (for reviews [1]–[6]). Major antigens on the iRBC surface include members of the *P. falciparum* erythrocyte membrane protein-1 (PfEMP1) family, highly polymorphic and modular proteins composed of DBL (Duffy binding-like) and CIDR (cysteine-rich interdomain region) domains [7]. On each iRBC, a single PfEMP1 is expressed from one of ∼sixty *var* genes [8],[9] in the parasite genome. A different *var* gene can be activated during each cycle of infection, resulting in a new surface PfEMP1 molecule, and the parasite's evasion of antibodies against the previously expressed PfEMP1 [10].

*Var* genes undergo frequent recombination and gene conversion giving rise in the parasite population to a vast number of variant PfEMP1 proteins [11] that are under immune system selective pressure. PfEMP1 molecules are thought to be constrained in sequence only to maintain binding to host receptors and thus to mediate cytoadherence [12]. Cytoadherence prevents iRBCs from passing through the spleen and thus being cleared from the circulation [13]. Adherence of iRBCs to uninfected RBC (known as rosetting) and adherence to the microvasculature (known as sequestration) are thought to be primary causes of morbidity, especially in cerebral and placental malaria. iRBCs adhere due to the binding of PfEMP1 to a variety of endothelial receptors among which are CD36, intercellular adhesion molecule-1 (ICAM-1), and chondroitin sulfate A (CSA). Almost all parasites isolated directly from patients have the capacity to bind to CD36 [14]. iRBC appear to modulate immune system responses by their binding to CD36 and to other receptors on immune cells in the blood [15]–[17].

**Figure 1:**
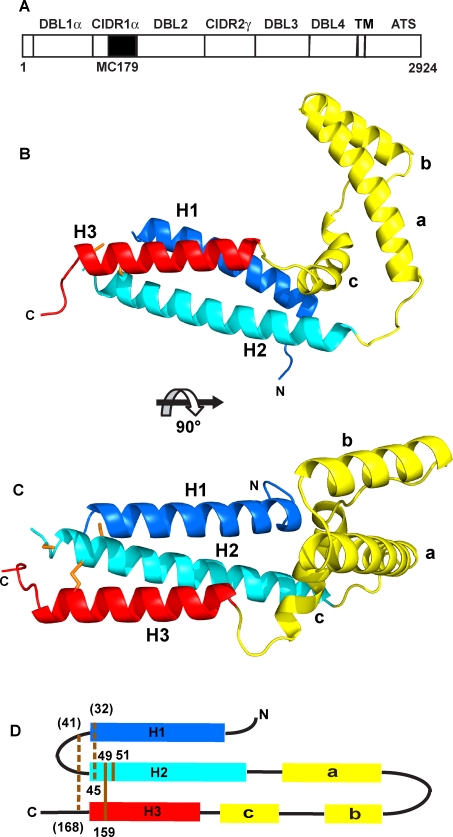
The MC179 structure and PfEMP1. (A) Two CIDR and four DBL domains compose the extracellular portion of the 2924-amino acid residue PfEMP1 molecule from the Malayan Camp (MC) strain of *P. falciparum* that is expressed on the surface of an infected red blood cell. MC179 (black box) is about 65% of the MC CIDR1α domain. Like most PfEMP1 molecules, the MC PfEMP1 has a semi-conserved N-terminal DBL1α-CIDR1α pair or “head structure” [7]. The transmembrane (TM) segment and acidic terminal segment (ATS) are located inside the infected red blood cell. (B) The MC179 structure is made up of three helices (H1, dark blue; H2, light blue; H3, red) that form a three-helix bundle and three additional helices (a, b, c; yellow) that connect H2 to H3. (C) View of MC179 after reorienting by 90°. Cysteine side chains are shown in orange. (D) MC179 topology diagram shows how the six helices are connected. Lines (orange) and residue numbers identify the positions of the three disulfide bonds and one unbonded cysteine. Two disulfide bonds were inferred to be present (dashed lines). Three cysteines (parentheses) were not observed in electron density. N- and C-termini (N, C) are labeled.

With the availability of genomic sequences from several *P. falciparum* strains, the numbers of DBL and CIDR domains and the extents of their diversity are becoming better known [18],[19]. Within the ∼sixty PfEMP1 sequences of the 3D7 strain of *P. falciparum*, there are 130 DBL and 110 CIDR domains [8],[20]. Though sequence identities among DBL and among CIDR domains are low, short blocks of conserved residues enable the classification of domains into families [21]. PfEMP1 domain families have been named DBLα, DBLβ, DBLγ, DBLδ, DBLε and CIDRα, CIDRβ, CIDRγ [18] and are also consistent with the groupings that arise from sequence similarities as performed by ClustalW [19]. Family groupings are used together with the position of the domain within its particular PfEMP1 to yield domain designations such as DBL1α, CIDR1α, CIDR2β, and so on. As an example, Figure 1A depicts the PfEMP1 molecule from the Malayan Camp (MC) strain of *P. falciparum* that consists of four DBL and two CIDR domains [22]. Correlations of domain families with specific ligand binding have been determined [23],[24]. In the case of the CIDR families, many domains have been tested for their binding to CD36. From these experiments, only CIDR1α domains bind CD36; other CIDR domains, even the closely related CIDR1α1 domains, do not [21],[25],[26].

Binding to CD36 by RBCs infected with the MC strain of *P. falciparum* has been studied [27],[28]. Experiments using labeled PfEMP1 proteins showed that a proteolytically-cleaved portion of PfEMP1 is capable of binding to CD36. Antibodies against a recombinant protein corresponding to residues 576 to 808 of the CIDR1α domain of MC PfEMP1 block adherence of iRBCs to CD36 [29]. Further analysis of recombinant portions of the MC PfEMP1 molecule identified a 179-residue polypeptide, MC179 (residues 576 to 754), as a minimal portion of the MC CIDR1α domain that retained binding to CD36 [30]. Recombinant MC179 competes with iRBCs for binding to CD36 under blood flow conditions, resulting in the release of bound RBCs [31],[32]. CIDR1α domains have been divided into three subdomains: M1, M2, and M3 [33]. MC179 corresponds to the M2 subdomain and M1 and M3 are the respective N-terminal and C-terminal portions of the CIDR1α domain. Vaccination with MC179 protects *Aotus* monkeys from severe infection upon challenge with the MC strain [34] and prevents severe anemia after infection with a heterologous strain [35].

Three-dimensional structures of DBL, but not CIDR domains, have been available, with sequence comparisons revealing a number of conserved hydrophobic residues and cysteines, yet with low overall sequence identities among PfEMP1 domains [7]. We present the structure and analysis of MC179 in the context of the broad sequence diversity of the CIDR domains of the *P. falciparum* genomes and indicate the insights into overall DBL and PfEMP1 structure that MC179 provides.

## Results/Discussion

### MC179 Structure and Binding to CD36

MC179 is composed of a bundle of three helices connected by loops and three additional helices (Figure 1). The first helix (H1) of the bundle is joined to the second helix (H2) through a disordered fourteen-residue loop. At the end of the H2 helix there are seventy residues in three smaller helices, **a**, **b**, and **c**, that form the connection to the third helix (H3). The “V-shape” of MC179 is made up of the three-helix bundle and the three helices between the H2 and H3 helices, in the order H1-H2-**a**-**b**-**c**-H3. The H1 and H2 helices make many contacts with each other and are linked by a conserved disulfide bond, Cys 32-Cys 45, that was not visible in the electron density, but inferred to be present. The H2 and H3 helices also make many contacts with each other and are linked by a conserved disulfide bond, Cys 49-Cys 159, that is observed in the electron density. The H1 and H3 helices, however, make only a few contacts within 4 Å distance and are not linked by a disulfide. The cysteine-rich area from which the domain gets its name is located near the end of the bundle where three disulfide bonds and one free cysteine are located (Figure 1D).

**Figure 2:**
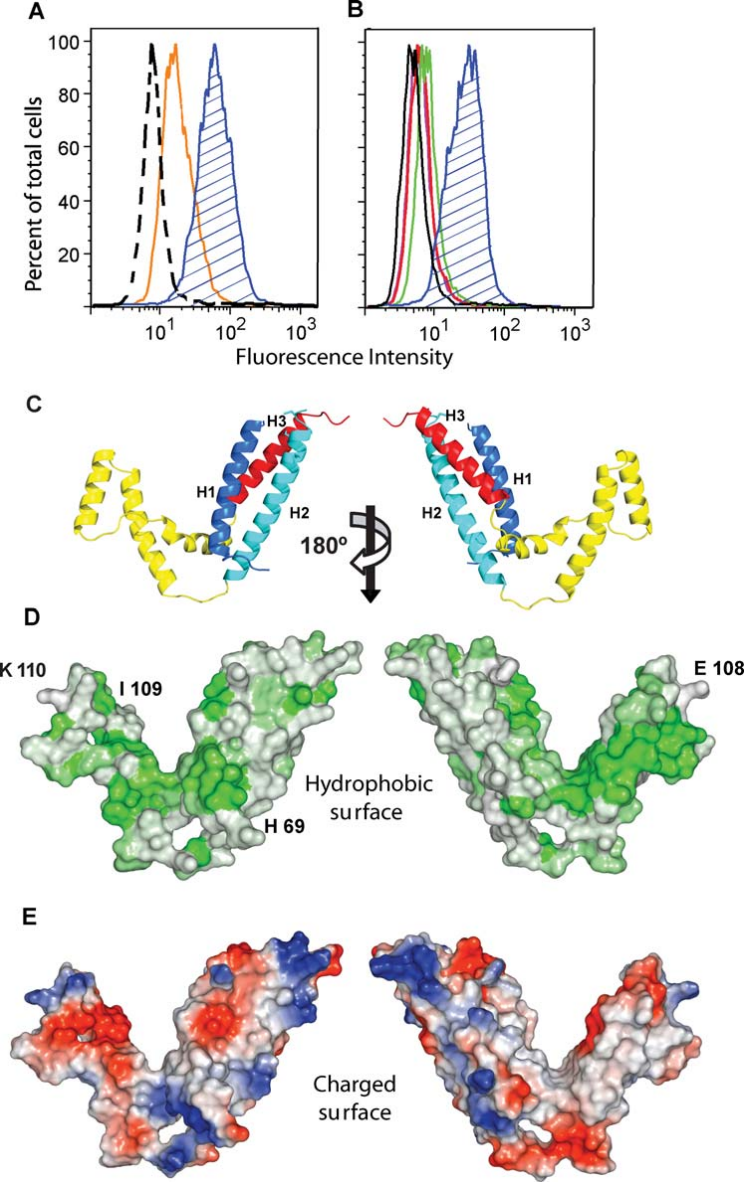
MC179 binding to CD36. Refolded MC179 bound to CD36-transfected CHO cells as assayed by flow cytometry ([A,B]; blue hatched peaks). (A) Incubation of MC179 with CD36-Fc fusion protein (100 µg/ml) diminished MC179 binding to CD36 on cells (orange peak). MC179 binding was revealed with anti-MC179 *Aotus* immune serum. (B) MC179 binding to CD36 is inhibited by each of three anti-CD36 mAbs (peaks outlined in red, violet, and green). The violet peak is under the red peak and is only partially visible. Binding of MC179 was visualized with an anti-pentaHis mAb. In both panels, controls used untransfected cells (peaks outlined in black). In (A), *Aotus* preimmune serum yielded results (not shown) identical to the untransfected control. (C) Ribbon diagrams of MC179 in the same orientations as (D) and (E). Three-helix bundles are located towards the center of the figure and connecting helices are at the sides. (D) Two views of the hydrophobic surface 180° apart show a hydrophobic patch (green, at right) on the connecting helices of MC179. Labeled residues are those reported to affect CD36-binding (see text). (E) Surface views of MC179 showing the distribution of positive (blue) and negative (red) surface charge. The negatively charged region (red, at left) on the connecting helices is on the opposite face from the hydrophobic patch at the right in (D).

Using mammalian cells transfected to express human CD36, we confirmed by flow cytometry that the MC179 portion of the CIDR1α domain that had been crystallized was capable of binding CD36. Adding MC179 to cells expressing CD36 revealed robust binding of MC179 (Figure 2A and 2B, blue hatched peaks). Preincubation of MC179 with soluble human CD36 produced in insect cells diminished the binding of MC179 to the CD36-expressing cells (Figure 2A, orange peak). This indicated that CD36 in solution could compete with cell surface CD36 for the binding of MC179. Preincubation of the CD36-expressing cells with anti-CD36 antibodies that are known to block the cytoadherence of parasitized RBC [36] prevented the binding of MC179 to CD36 (Figure 2B, green, red, violet peaks). This result signified that the binding of cytoadherence-blocking antibodies to CD36 could interfere with the binding of MC179 to CD36.

### The Cysteines of MC179

The cysteine-richness for which the domain is named stems from the conserved cysteines located on the H1-H2 loop, at the N-terminus of H2, and at the C-terminus of H3 (Figure 1D). We directly observed four of the seven cysteines and have evidence that six of the seven cysteines in MC179 are in disulfide bonds. The disulfide bond Cys 49-Cys 159 between the H2 and H3 helices was observed in electron density. Two other cysteines (Cys 45 and Cys 51) at the N-terminal end of the H2 helix were also observed in electron density. Difference electron density based on the sulfur anomalous signal in the native X-ray dataset confirmed the observation of each of the four cysteines. The sulfur atoms of cysteines 32 and 41 on the loop between the H1 and H2 helices and of cysteine 168 at the C-terminus of MC179 were not visible.

During the preparation of MC179 for crystallization, mass spectrometry detected an adduct of the size of a cysteamine molecule on MC179. We reasoned that one cysteine had reacted with cystamine (the oxidized form of cysteamine) during protein refolding, which implied that there was one free cysteine in MC179. From comparisons with DBL domains, we concluded that Cys 51 is the single unpaired cysteine in MC179 (see below). In experiments to determine which cysteine possessed the adduct, we deleted Cys 168 by truncating twelve residues from the MC179 C-terminus, resulting in the shorter MC167 construct. After refolding, mass spectrometry of MC167 showed that two cysteines had reacted with cystamine, indicating that the deletion of Cys 168 had generated a second free cysteine. This implied that Cys 168 in MC179 participates in a disulfide bond. Reports that MC179 with Cys 168 substituted by serine and a MC167-like truncated molecule still bind CD36 [30],[37] encouraged us to produce MC167 crystals and to use them interchangeably with MC179 during the structure determination. As residues 168–179 and the loop between H1 and H2 were not visible in the MC179 structure (see Methods), the observed X-ray structures of MC167 and MC179 are identical.

### Mapping Mutations and Their Effects on CD36 Binding

How do MC179 and thus the CIDR1α domains of PfEMP1 molecules bind CD36? The effects of mutations chosen based on sequence similarities in several regions of MC179 have been reported and these mutations can now be located and defined structurally. The substitution of Ser for Cys 45 or a double substitution of Ser for both Cys 159 and Cys 168 abolished CD36 binding [30]. Except for those cysteines, point mutations have little effect on CD36 binding, but changing many residues at the same time has a large effect on CD36-binding. CD36-binding was not affected when point mutations within helices H1 and H2 of the three-helix bundle were made or when the residues between Cys 32 and Cys 41 on the loop connecting the H1 and H2 helices were replaced with residues from a non-binding CIDR domain [33]. When several point mutations were placed into the same molecule, CD36 binding diminished from 100% to 63% [21]. CD36-binding was lost when chimeric proteins were made between the MC CIDR1α domain and CIDR domains that do not bind CD36. When the H1-H2, **a**-**b**-**c**, and **a**-**b**-**c**-H3 regions of MC179 were substituted in turn with the corresponding sequences from the non-binding CIDR domains, no binding to CD36 was detected [21],[33]. The MC179 domain appears to function as a whole in binding CD36 and only large changes in the domain have succeeded in affecting binding to CD36.

Three point mutations in the same molecule affected the binding of several CIDR1α domains to CD36. The wild type CIDR1α domain from the ItG2-CS2 parasite strain does not bind to CD36, but spontaneous mutations that arose during in vitro culture changed each of the residues in the sequence Gly-His-Arg, resulting in CD36-binding [33]. Sequence comparison aligns Gly-His-Arg with the MC179 sequence, Glu 108-Ile 109-Lys 110, which is located at the beginning of the **b** helix and makes contact with the end of the **a** helix (Figure 2D). Glu 108 lies just above a hydrophobic patch in the right-hand panel of Figure 2D. Replacing Glu-Ile-Lys with the wild type ItG2-CS2 Gly-His-Arg sequence decreased MC179 binding to CD36 by 50% [33]. This suggests that part of the CD36-binding region of MC179 lies within or near the **a** and **b** connecting helices.

Recently, a chimeric protein (“1640-f”) between a non-CD36-binding CIDR1α1 and a CD36-binding CIDR1α molecule exhibited CD36 binding [25]. Based on sequence alignments (Figure S2) and the MC179 structure, the 1640-f chimera contained the H1, H2, **a**, and **b** helices from the CIDR1α1 (PFE1640w) domain and the **b**, **c**, and H3 helices from the CIDR1α (PF10_0406) domain. It appears that the 1640-f chimera contained two copies of the **b** helix. Antibodies raised against the CIDR1α portion of the chimera inhibited CD36 binding by erythrocytes infected with the 3D7, HB3, or FCR3 parasite strains, which indicated that there are similar epitopes on CIDR domains that otherwise differ from each other [25]. Where might these cross-reactive epitopes be located? The **c** helix has few conserved residues and the conserved residues of the **b** helix point inward toward the **a** helix, though they still could be contacted by antibodies. We suggest that the H3 helix may be the location of cross-reactive epitopes since the side chains of some conserved residues of the H3 helix, such as Asp 146 and His 151, extend out into solvent.

Sequences and lengths of the **a** and **b** helices in CD36-binding CIDR1α domains vary substantially (Figure S1), which is difficult to rationalize with CIDR1α binding to the non-polymorphic CD36 protein. In addition, mutations and chimeric molecules have implicated several areas of the MC179 molecule in binding. An understanding of the mechanism of binding CD36 has not been obtained through mutational analysis of conserved residues. That hundreds of CIDR1α sequences still bind CD36 while containing multiple “natural” mutations implies that CD36-binding may derive from the overall structure of the CIDR1α domain.

### A Surface Conserved in Both CD36 Binders and Non-Binders

**Figure 3:**
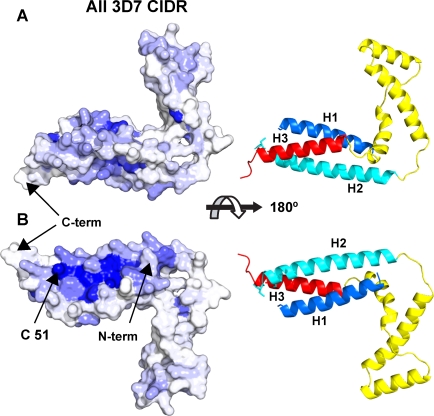
Sequence conservation of all CIDR domains of the 3D7 genome mapped on to the MC179 structure. High to low conservation is mapped as a color gradient (dark blue to white) on the molecular surface of MC179. A highly conserved surface patch (dark blue) means that a high percentage of the residues at that position in the alignment of all 3D7 CIDR domains (Figure S2) are identical to the surface-exposed MC179 residue. (A) View of one side of MC179 reveals little conservation. (B) View after a rotation of 180° shows a highly conserved region on the molecule. Cys 51 is conserved in all CIDR domains. Other highly conserved residues that contribute to the conserved surface between Cys 51 and the N-terminus are Trp 14, Asp 21, Trp 55, Lys 59, Glu 62, and Ile 66 (not shown). Little conservation is seen on the surfaces of the connecting helices. The cartoons to the right are reference diagrams in the same orientations as the surfaces. A few highly conserved residues are not visible at the surface because they are completely buried. Sequence alignments (Figures S1, S2 and S3) were used as input to the Protskin server [38] to produce this figure and Figure 4.

We aligned the MC179-like sequences of the 110 CIDR domains of the *P. falciparum* 3D7 genome (Figure S2). For each position in the MC179 sequence, the percent of aligned sequences having the identical residue at that position was calculated [38]. We plotted the percent sequence identity as a gradient of color (blue) on a surface depiction of MC179 (Figure 3). This revealed a conserved region found in all CIDR domains (Figure 3B). This region is located between the H1 and H2 helices near to the N- and C-termini of MC179 and contains the unpaired cysteine, Cys 51 (Figure 3B). Cys 51 is located on the H2 helix and is conserved in all CIDR domains whether CIDR1 or CIDR2 (Figure S2).

The conserved surface shown in Figure 3B likely becomes part of the interior of the CIDR domain in the presence of the M1 subdomain of CIDR1α that is located N-terminal to the MC179 or M2 subdomain [37]. In other words, the blue surface in Figure 3B is predicted to be the interface between the M1 and M2 subdomains because it is conserved in all CIDR domains, is proximal to the MC179 N-terminus, and contains the unpaired, conserved Cys 51. In the intact CIDR domain, Cys 51 is likely disulfide-bonded to one of the cysteines of M1. The N-terminal part of MC179 was shown to be important in binding CD36 [30] and MC179-analogous protein constructs from some *P. falciparum* strains bound CD36 only after the polypeptides were lengthened to include M1 [37].

Examination of the MC179 molecule reveals that the surface on the opposite face of MC179 is little conserved (Figure 3A). The surfaces of the **a**, **b**, and **c** helices are not conserved (Figure 3) and the connecting helices in CIDR1α sequences vary in length. Therefore, a large part of the MC179 surface is not conserved, as most of the residues making up the surface of MC179 are not conserved in other CIDR1α domains. We propose that much of MC179 is exposed at the surface of the PfEMP1 molecule. Of course, MC179 must be exposed at the surface to bind CD36. How much of it? Perhaps 1000 Å^2^ would be a minimum estimate in light of other known protein-protein interactions. MC179 has a total solvent accessible area of 10,000 Å^2^. Therefore, at least 10% of MC179 is exposed. We detected conserved patches that we attribute to the interface with the M1 subdomain of CIDR1α and to potential CD36 binding (see below). We did not detect a conserved surface that might be an interface with DBL domains, for instance, DBL1α or DBL2, although the M1 portion may provide a conserved contact surface with DBL1α. It seems likely to us that the CIDR1α domain has only a small surface area in contact with the DBL domains and that more than 10% of the area of MC179 is exposed to the bloodstream. MC179 likely extends out from the PfEMP1 molecule, consistent with the proximity of the MC179 N- and C-termini to the conserved patch predicted to interface with the M1 portion. Persons living in malaria endemic regions only slowly acquire effective antibodies against iRBCs [39]. One or more variable MC179-like surfaces on PfEMP1 molecules would provide a high diversity of antibody epitopes, leading to the slow development of immunity and to the parasite's ability to evade the host immune response.

### A Second Conserved Surface on CIDR1α Domains

**Figure 4:**
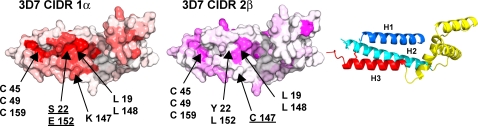
Comparison of CIDR1α and CIDR2β conserved surfaces. Sequence conservation (Figures S1 and S3) has been mapped as in Figure 3. MC179 has been rotated about 90° from the orientation in Figure 3 to show the region between the H1 and H3 helices. At left, a bright red patch means that a high percentage of 3D7 CIDR1α domains contain the same residue at the particular position as does MC179. In the middle, a bright violet patch means that a high percentage of 3D7 CIDR2β domains contain the same residue at the particular position as does MC179. Appearing at the surface of both types of domain are Cys 45, Cys 49, and Cys 159 that form part of the conserved disulfide bond network and Leu 19 and Leu 148 that make a conserved interaction between H1 and H3. The unusual nearly buried Ser 22-Glu 152 pair is unique to CIDR1α domains. At position 147, a conserved lysine is found in CIDR1α domains, but is a cysteine in most CIDR2β domains. At right is a reference diagram for the orientation of MC179 in this figure.

The majority of PfEMP1 molecules from the 3D7 parasite strain contain one CIDR1α domain that binds CD36 and one CIDR2β domain that does not bind CD36 [21]. We mapped the percent identity of the MC179 sequence with the 3D7 CIDR1α domains (Figure S1) and with the 3D7 CIDR2β domains (Figure S3) on separate surface models of MC179 (Figure 4). This revealed a conserved region located between the H1 and H3 helices that is present in both kinds of domains, but is larger in CIDR1α domains. Every CIDR domain has some identity with MC179 here, accounted for by conserved residues that make up the interior of the three-helix bundle, but are partially exposed at the surface of the molecule. Examples of these are three cysteines (45, 49, and 159) and two leucines (19 and 148) (Figure 4).

Contributing to this surface in CIDR1α domains are a conserved serine and a conserved glutamic acid. In MC179, Ser 22 and the charged Glu 152 are both nearly buried and form a hydrogen bond. Except in one PfEMP1 (PFL0020w) where Ser 22 is replaced by a cysteine residue, Ser 22 and Glu 152 are completely conserved in CIDR1α domains, but one or both of the residues are absent in other CIDR domains. Ser 22 becomes a tyrosine in most other CIDR domains and Glu 152 becomes a leucine in all other 3D7 CIDR domains (Figure 4, middle). Although this conserved surface in CIDR1α domains with a nearly buried glutamic acid residue might be a potential interaction surface with CD36, mutations of Ser 22 to threonine [33] and Glu 152 to leucine [21] have not affected CD36 binding. Additionally, Lys 147 is present in most CIDR1α sequences, within the conserved region shown in Figure 4. In almost all CIDR2β domains, the residue at the Lys 147 position is a cysteine (Figure 4).

### CIDR Domains Are Structurally Related to DBL Domains

**Figure 5:**
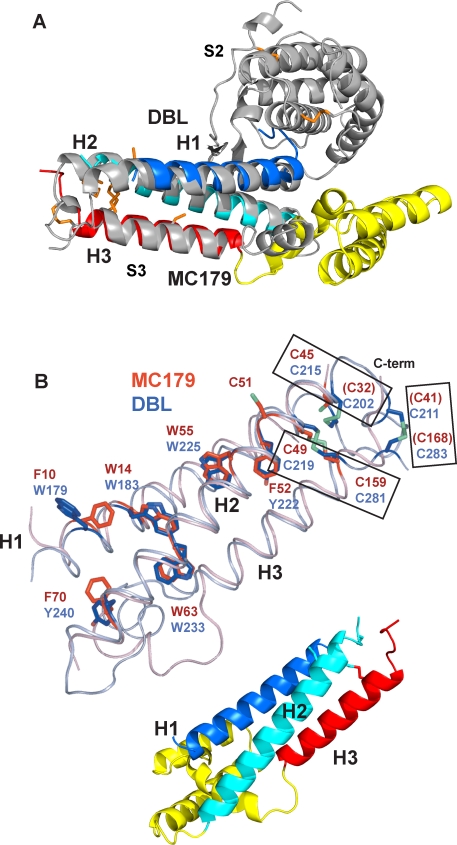
CIDR and DBL domains have structurally identical three-helix bundles. (A) The three-helix bundle of MC179 closely overlays the C-terminal three-helix bundle of the F1 DBL domain of EBA-175 (gray). MC179 superimposes similarly on the F2 DBL domain of EBA-175 and the Pkα-DBL domain (not shown). Note that the connecting helices (yellow) extend between H2 and H3, but the DBL subdomain–subdomain interaction is approximately 120° away between the H1 and H2 helices. This overlay models our prediction of the DBL1α C-terminal three-helix bundle, with the yellow connecting helices of MC179 modeling the predicted connecting helices of DBL1α (see text). DBL subdomain 2 (S2) and subdomain 3 (S3) are labeled [41]. (B) The three-helix bundles of MC179 (pink helices) and of the F1 DBL domain (steel blue helices) are superimposed, after an approximate 180° reorientation from (A) about the vertical axis. The connecting helices between H2 and H3 are not shown for clarity. Note the almost identical positions of the helices and of conserved Phe, Trp, Tyr, and Cys side chains (red, MC179; blue, F1 DBL). Cysteines making conserved disulfide bonds (rectangles) are from MC179 (red font) and from the F1 DBL domain (blue font). Parentheses enclose the three cysteines not observed in the MC179 electron density. Cartoon diagram serves as a reference for the orientation of MC179 in (B).

**Figure 6:**
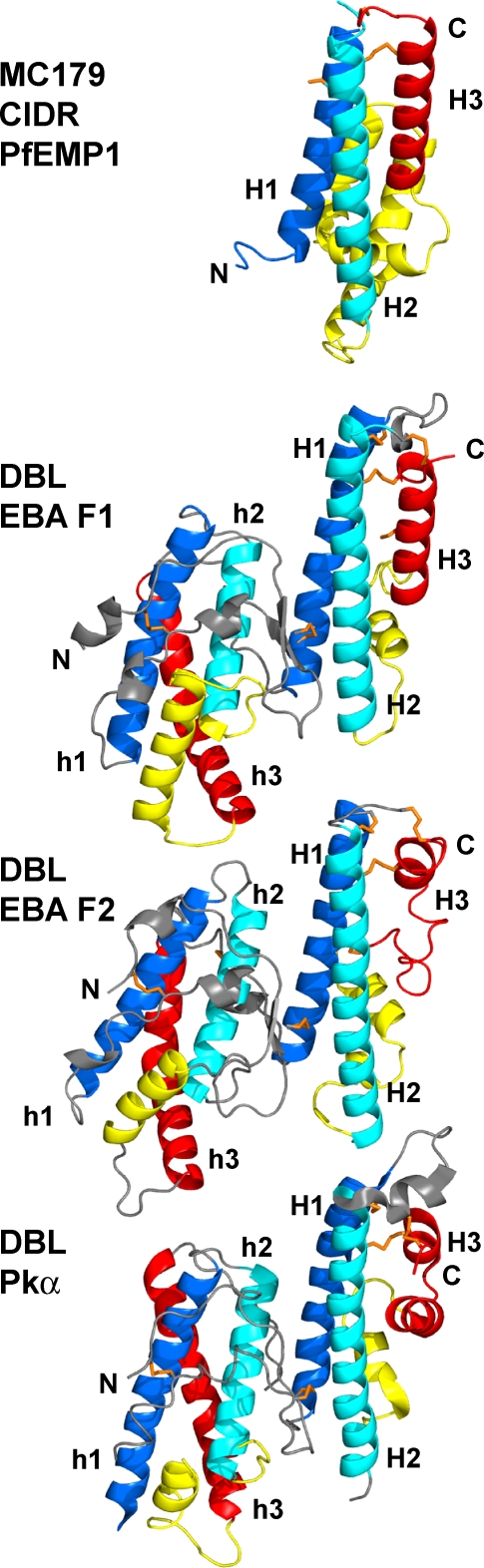
The three-helix bundles of MC179 and the DBL domains. All four structures were aligned using the alpha carbon atoms of the H1 and H2 helices, then were translated apart for viewing. In subdomain 3, the similarity of the H1 (dark blue) and H2 (light blue) helices is apparent, but the H3 (red) helices differ among molecules. MC179 and the EBA-175 F1 DBL each contain regular H3 helices, but the F2 and Pkα DBL domains have irregular H3 helices. Nevertheless, these degenerate helices meander to a pair of conserved cysteines that make conserved bonds to H1 and H2, as in MC179 and in the F1 domain. DBL domains contain another three-helix bundle (h1, h2, h3) in the N-terminal half (subdomain 2) of the molecule that is colored here analogously to the MC179 H1, H2, H3, and connecting helices. Several characteristics of these N-terminal bundles suggest their relatedness to the MC179 bundle and to the C-terminal DBL bundles (see text). N- and C-termini (N, C) are labeled.

During the first cloning and sequencing of PfEMP1 genes, conserved hydrophobic residues and cysteines observed in the midst of the enormous sequence diversity of these large proteins were used to define the DBL and CIDR domains and to suggest their relatedness [7]. Recent crystal structures of non-PfEMP1 DBL domains [40],[41] from the *P. falciparum* erythrocyte-binding antigen (EBA)-175, containing tandem DBL domains F1 and F2, and the *P. knowlesi* erythrocyte binding protein (Pkα-DBL), containing a single DBL domain, showed that conserved residues are within DBL helices. We superimposed the MC179 structure on the F1 DBL domain from EBA-175 (Figure 5A), the F2 DBL domain from EBA-175, and the DBL domain from Pkα (Figure 6). The three-helix bundle of MC179 structurally matched each DBL C-terminal three-helix bundle, a region that has also been named “subdomain 3” [41]. MC179 and all three DBL structures closely overlaid with root mean squared (r.m.s.) deviations between 1.6–2.0 Å over the 74–77 α-carbon atoms of the three helices. The overlay is best among the H1 and H2 helices of all four proteins and between the regular H3 helices of MC179 and the EBA-175 F1 DBL domain. The H3 helices of EBA-175 F2 and of Pkα-DBL are mixed with irregular secondary structure and take a meandering route along their respective H1 and H2 helices. This structurally conserved three-helix “bundle” is a feature of the DBL and CIDR protein folds and is evidence beyond their sequence similarities for the common ancestry between the two domain families [7].

More than main chain atoms match among the structures, however, as even the conserved side chains in the H1, H2, H3 helices of MC179 and in the corresponding helices of the DBL domains are in identical positions. In Figure 5B, six conserved Trp, Phe, and Tyr side chains in the H1 and H2 helices closely overlay between MC179 and the F1 DBL domain. In addition, the Cys 49-Cys 159 disulfide and Cys 45 side chain of MC179 overlay to within 3.0 Å of the analogous cysteines of the EBA-175 and Pkα-DBL domains. It is clear that the MC179 and thus the CIDR1α disulfide bonding is the same as in the DBL domains (Figure 1D and 5B). In the MC179 numbering used in the structure, the cysteines involved in disulfide bonds are 32–45, 41–168, and 49–159 (Figure 5B). Cys 51 extends into solvent from the H2 helix and is conserved in all CIDR, but not DBL domains. Cysteines 49 and 51 form the Cys-X-Cys sequence motif that is found in most CIDR sequences [18]. Sequence alignments and more recently, detailed modeling studies based on the EBA-175 and Pkα crystal structures, have predicted that the interiors of DBL and CIDR domains would be held together by conserved hydrophobic and cysteine residues and that loops would contain the most polymorphic sequences [42],[43].

The structural overlay of MC179 on the available DBL domains also reveals the differences in the connections between the helices of the bundle (Figure 6). A loop of approximately similar length connects the H1 and H2 helices in both MC179 and in the structurally known DBL domains, but the connection between the H2 and H3 helices differs considerably between the two kinds of domains. In the known DBL domains, the H2-H3 connections are about 30 residues in length consisting of a small loop and helix that vary among the three DBL domains. In MC179, the H2-H3 connection is about 70 residues in length and ranges from 30 to 100 residues in the many known CIDR sequences.

### Two Three-helix Bundles in DBL Domains

While examining the similarities between MC179 and the known DBL domains, it became evident that DBL domains are composed of two three-helix bundles (Figure 6). As described above, MC179 structurally matches the DBL C-terminal three-helix subdomain, but the DBL subdomain nearer to the N-terminus is also made up of three helices that are connected like the MC179 and C-terminal DBL bundles. The sequence extents of the N-terminal and C-terminal three-helix bundles in the structurally determined DBL domains are: residues 58–159 and 174–282 in EBA-175 F1, residues 365–463 and 481–596 in EBA-175 F2, residues 64–165 and 190–304 in Pkα-DBL (PDB codes 1ZRL and 2C6J). In Figure 6, MC179 and the three DBL domains are colored to highlight bundle helices and connections between helices. MC179 structurally overlays on the DBL C-terminal three-helix bundle, but does not overlay on the N-terminal bundle. However, the helices and the connections between the helices of the N-terminal bundle (h1, h2, h3) are similar to those of the C-terminal bundle: the h1 helix (dark blue), a short connecting loop (gray), the h2 helix (light blue), a longer connecting helix (yellow), and the h3 helix (red) (Figure 6). Like the C-terminal bundle, the N-terminal bundle contains a disulfide that connects the h2 helix at its N-terminus with the h3 helix at its C-terminus. The cysteines that compose this disulfide in each DBL domain are: Cys 88-Cys 164 in EBA-175 F1; Cys 396-Cys 471 in EBA-175 F2; Cys 99-Cys 176 in Pkα-DBL. These disulfides pin the h2 and h3 helices to each other and correspond to the Cys 49-Cys 159 disulfide of MC179 and the analogous disulfides of the C-terminal three-helix bundles of DBL domains.

### Locating the H1 and H2 Helices

**Figure 7:**
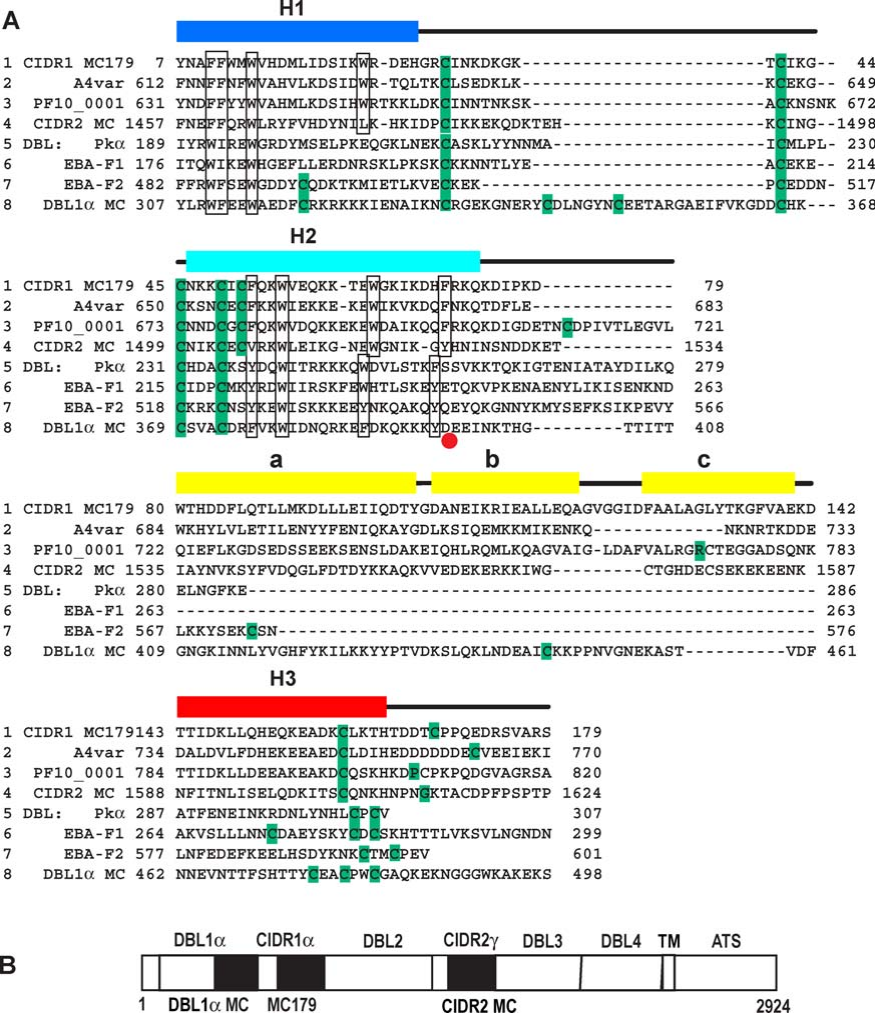
Sequence comparison of MC179, CIDR, and DBL domains. (A) Alignment of four CIDR and four DBL domains with the lines numbered 1–8 for clarity of discussion. Lines 1–4 show MC179 and the MC179-like portions of three other CIDR domains: line 1, CIDR1α MC179; line 2, CIDR1α of A4var PfEMP1; line 3, CIDR1α of PF10_0001 of 3D7; and line 4, CIDR2γ of the MC strain (see [B]). Lines 5–8 show four DBL domains: line 5, Pkα; line 6, EBA-F1; line 7, EBA-F2; and line 8, DBL1α of MC PfEMP1. The sequence alignment is based on conserved cysteines (green), conserved hydrophobic residues (boxed), and on superimposed X-ray structures. Note the differing lengths and high sequence diversity in regions of lines 2–8 that are aligned with the a, b, and c helices (yellow) that connect H2 and H3 of MC179. The short connection between the H2 and H3 helices in the non-PfEMP1 DBL domains (lines 5–7) contrasts with the much longer connection observed in the MC179 structure (line 1), predicted in other CIDR domains (lines 2–4), and predicted in DBL1α (line 8). Note the long loop in DBL1α that connects the H1 and H2 helices. The end of the H2 helix is marked (red dot). Helices from the MC179 structure are positioned over the MC179 sequence in line 1. MC179 is numbered 1–179 based on the PDB accession code 3C64, which is the same sequence as residues 576–754 of GenBank U27338. The other sequences are numbered as in their GenBank or PDB depositions with accession codes: A4var, L42244; PF10_0001; CIDR2 MC, U27338; Pkα, 2C6L; EBA-F1, 1ZRO; EBA-F2, 1ZRO; DBL1α MC, U27338. (B) Diagram of the entire PfEMP1 from the MC strain showing the alternating DBL and CIDR domains as in Figure 1. Filled boxes denote locations of the “DBL1α MC,” “CIDR1 MC179,” and “CIDR2 MC” sequences that are aligned in (A).

Based on structural similarities and conserved residues among the CIDR and DBL domains, we developed sequence expressions to detect the H1 and H2 helices of the three-helix bundles that can be used to locate CIDR and DBL domains in PfEMP1 sequences. These sequence patterns describe the conserved residues that are unique to the H1 and H2 helices. The H1 helix sequence of CIDR and DBL domains satisfies the expression: (Leu, Phe, Trp, or Tyr)-X_3_-Trp-X_17, 18, or 19_-Cys, where “X_subscript_” indicates the number of residues of unspecified type occurring between conserved residues. Several possible hydrophobic residues are indicated in parentheses at some positions. As examples, the H1 helix of MC179 is located with the pattern Phe-X_3_-Trp-X_17_-Cys and the predicted H1 helix of the Malayan Camp DBL1α is found with Trp-X_3_-Trp-X_18_-Cys (Figure 7).

Different patterns of conserved residues distinguish the CIDR H2 helices and the DBL H2 helices. The H2 helix is found in CIDR domains by the expression: Cys-X_3_-Trp-X_7 or 8_-Trp-X_6_-(Phe or Tyr). This motif is preceeded by an additional Cys-X_1_- in many CIDR domains. In DBL domains, H2 helices are predicted with this algorithm: Cys-X_3_-Cys-X_2_-(Phe or Tyr)-X_2_-(Leu, Lys, Trp, Tyr)-X_7_-(Phe, Trp, or Tyr)-X_6_-(Phe or Tyr).

We used the CIDR algorithms for H1 and H2 to detect a CIDR-like domain in the *var2csa* protein, the product of a relatively conserved gene found in all *P. falciparum* strains and expressed by most parasites that bind CSA [44],[45]. For example, the *var2csa* family member in the 3D7 genome, PFL0030c, contains a CIDR-like domain between the DBL2x and DBL3x domains, two of the six DBL domains of PFL0030c. The H1 pattern of this CIDR-like domain is Leu-X_3_-Trp-X_18_-Cys and the H2 pattern is Cys-X_3_-Trp-X_7_-Trp-X_6_-Tyr. We predict a three-helix bundle in this CIDR-like domain that extends from residues 1025 to 1212 of the PFL0030c protein. This CIDR-like domain, termed the ID (interdomain)-2 domain, has recently also been predicted through sequence analysis and modeling [43].

### MC179 Forms a Dimer in the Crystal

**Figure 8:**
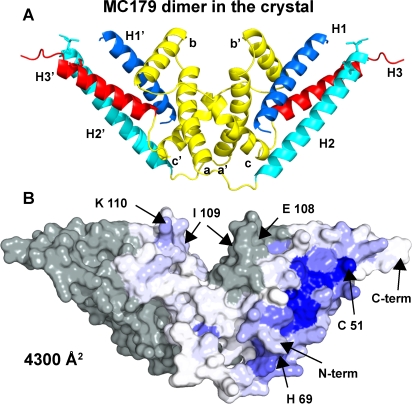
MC179 forms a dimer in the crystal. (A) Contact between the molecules is primarily between the helices (yellow) that connect the H2 and H3 helices and between the H1 (dark blue) to H3 (red) sides of the three-helix bundles. (B) The molecular surface of the crystalline dimer shows the intertwining of the two molecules in a “handshake” manner. One molecule (at right) is colored by sequence conservation in a ramp of blue to white as in Figure 3, and the other molecule (at left) is colored gray for contrast. The total buried solvent-accessible surface area is 4300 Å^2^. The surface complementarity is 0.70 [46] and 55 residues (220 atoms) from each molecule take part in the interaction. The two MC179 molecules are related by a crystallographic two-fold symmetry axis. The locations of several residues are indicated.

In the MC179 crystal, pairs of molecules interact and bury 4300 Å^2^ of solvent accessible surface area between them (Figure 8). A crystallographic two-fold symmetry axis relates the two MC179 molecules. Much of the contact between the molecules is made by the helices that connect the H2 and H3 helices including the hydrophobic patch seen in Figure 2D. Helix **a** residues (Leu 94, Leu 95, Leu 96, Ile 98, Ile 99) and helix **b** residues (Ile 112, Leu 115, Leu 116) constitute this hydrophobic area on one molecule, interacting with H1 helix residues (Trp 12, Val 15, Leu 19, Ile 20, Ile 23) and H3 helix residues (Thr 144, Lys 147, His 151) of the symmetry-related MC179 molecule. Hydrophobic areas in the third connecting helix, helix **c**, also contribute to this interaction between molecules in the crystal. Opposite to the hydrophobic patch on the **a**, **b**, and **c** helices is a region made up of negative charges from helix **a** residues (Glu 97, Asp 101), helix **b** residues (Glu 113, Glu 117), and a helix **c** residue (Asp 125) (Figure 2E, left side in red). These charges are on the surface of the crystallographic dimer. The molecular surface of the crystalline dimer shows the intertwining of the two molecules in a “handshake”-like interaction (Figure 8B). The surface complementarity (SC) index of the monomer-monomer interface is high at 0.70 [46] and 55 residues (220 atoms) from each molecule take part in the interaction. Residues that when mutated have affected CD36 binding (His 69, Glu 108, Ile 109, Lys 110) contribute to the surface of the MC179 dimer and, except for His 69, are located near, but not in, the dimer interface (Figure 8B). His 69 is distant from the dimer interface.

The interaction between MC179 molecules in the crystal resembles a biological interaction with its large amount of buried surface area and high surface complementarity. In solution, when refolded MC179 was prepared by size exclusion chromatography, we separated molecular species with the mobility of dimers from the desired species with the mobility of monomers and used the monomers for crystallization. In addition, chemical cross-linking experiments indicated the presence of MC179 dimers in solution (data not shown). We conclude that MC179 has the tendency to dimerize in solution. How these dimers of a recombinant fragment of PfEMP1, if biologically relevant, might function in a PfEMP1 molecule is unclear. Based on the enormous contact area observed in the MC179 “dimer” in the crystal, our hypothesis is that the connecting helices of the MC179 portion of CIDR1α domains bind another molecule, either a PfEMP1 domain or a ligand. This hypothesis that the CIDR connecting helices are involved in binding another molecule extends to our prediction of binding by the proposed connecting helices of the DBL1α domain as described below.

### Examination of CIDR1α Sequences

Using BLAST with the MC179 amino acid sequence as probe, we aligned the MC179 portions of the approximately 300 CIDR1α sequences from the 3D7 strain, the Ghanaian clinical isolate, the IT strain, and *P. reichenowi* genomes from the Sanger sequencing center (http://www.sanger.ac.uk/Projects/Protozoa/). In the alignment, semi-conserved hydrophobic residues implied the presence of the MC179 connecting helices **a**, **b**, and **c**. By their sequences, all CIDR1α domains appear to retain approximately the first two connecting helices, the **a** and **b** helices, as predicted by alignment with the MC179 sequence, but the third connecting helix **c** varies greatly in length (see 3D7 CIDR1α alignment in Figure S1). These differing CIDR1α sequences and lengths must result in changes in the size of the V-like opening of the CIDR1α domain as seen in the MC179 structure. The sequence composition and length of the three connecting helices (**a**, **b**, and **c**) vary even among CIDR1α molecules that bind CD36 (Figure S1). In some CIDR1α sequences there are two additional cysteine residues, one located about ten residues after the end of the H2 helix and the other located about ten residues before the start of the H3 helix. In the MC179 structure, these two Cα atom positions are separated by about 8 Å, which could be spanned by a disulfide bond. For example, PF10_0001 (Figure 7), PFD0630c, PFD0005w from the 3D7 strain have such cysteines. We predict that these cysteines form a disulfide bond that could act to stabilize the MC179 structure, by linking the **a** and **b** helices. Such a disulfide would prevent movement of helices **a** and **b** relative to each other, which would need to be considered in models of how CIDR1α binds CD36, as PF10_0001, PFD0630c, and PFD0005w are CD36 binders [21].

### Insights into DBL1α Domains of PfEMP1 Molecules

Comparisons of the MC179 and DBL X-ray structures with DBL1α domain sequences indicate that DBL1α domains have a longer H1-H2 connecting loop than do the MC179 and DBL domains. Figure 7 presents the DBL1α domain of the MC PfEMP1 as an example. DBL1α domains also have an additional cysteine in the H1 helix and two additional cysteines within the connecting loop to H2 (Figure 7). The presence of extra cysteines in PfEMP1 DBL domains has complicated the identification of conserved cysteines. The MC179 structure will aid in the comparison of PfEMP1 DBL, non-PfEMP1 DBL, and CIDR domains.

From the current knowledge of the MC179 and DBL X-ray structures, it is clear that the C-terminal boundary of DBL1α domains has sometimes been predicted to be at the end of the H2 helix of the three-helix bundle, as determined from conserved residues and the number of residues between them (Figure 7). This is understandable since after the end of the H2 helix there is little sequence similarity among DBL domains to guide prediction. The MC179 and DBL structures show that a third helix, degenerate helix, or, conceivably, an irregular H3 structure must be present in all DBL and CIDR domains to complete the “bundle”. The structures also predict that there are at least two cysteines at the C-terminus of the third helix that make conserved bonds with Cys 41 and Cys 49 (MC179) or their homologs in the DBL domains (Figures 5B and 7A).

This need for two cysteines at the end of the DBL domain can be used to predict the C-terminus of the DBL1α domain and leads to the prediction of MC179-like connecting helices in DBL1α. For example, in the Malayan Camp sequence shown in Figure 7A (line 8, DBL1α MC), if one proceeds along the DBL1α MC sequence from the end of the H2 helix at conserved residue Tyr 394 (Figure 7A, red dot), a single cysteine is encountered at residue 445, but a cluster of three cysteines is found at residue 475 (Figure 7A, DBL1α MC). Since two cysteines are required to form disulfide bonds with two conserved cysteines near the start of the H2 helix, the third helix H3 of the DBL1α must include the multiple cysteines around residue 475 in the sequence -Cys-Glu-Ala-Cys-Pro-Trp-Cys- (-CEACPWC-). If the DBL1α domain has a three-helix bundle that ends around position 475, then the length of the three-helix region is about 70–80 residues longer than in the EBA-175 and Pkα-DBL domains. This leads to the prediction that the DBL1α domain likely has connecting helices between H2 and H3 of about the same length (70–80 residues) as does MC179 (Figure 7).

The definition of a longer DBL1α domain affects the predicted start of the following CIDR1α domain. Due to the longer DBL1α domain, the immediately following CIDR1α domain and its M1 region are predicted to be 70–80 residues shorter. This reasoning implies that the DBL1α C-terminal region has been included in some predictions of the CIDR1α domain. Locating the DBL domain C-terminal boundary in this way by finding the cysteines that end the third helix is applicable to the DBL sequence classes, α, β, γ, δ, ε, and to the analysis of the semi-conserved domain pairings of DBL1α-CIDR1α, DBL2β-C2, and DBL2δ-CIDR2β that frequently appear in PfEMP1 sequences [18].

The DBL1α-CIDR1α domain pairing or “conserved head structure” is present at the N-terminal end of a majority of PfEMP1 molecules [7],[8]. As the sequences that connect H2 to H3 in the DBL1α and CIDR1α domains of the MC strain appeared to be similar in length, we examined the lengths of connecting regions in all of the DBL1α and CIDR1α domains that are pairs in the 3D7 genome. We counted the number of residues that connect H2 and H3 for each DBL1α and its associated CIDR1α domain and produced a list of forty-seven pairs of numbers, one pair of numbers from each PfEMP1 with a conserved head structure. Each number is the length of the connecting sequence in a DBL1α or a CIDR1α domain. The lengths in the list are correlated with a 0.58 correlation coefficient (Spearman rank-order) between them (p<0.0001) (Figure S4). This positive length correlation between DBL1α and CIDR1α implies that the connecting sequences of the DBL1α domains are similar in length to the CIDR1α connecting regions. This may simply reflect the evolutionary relatedness of the two domains, but may indicate similar binding or other function.

The H2-H3 connecting helices of DBL1α may extend away from the PfEMP1 molecule, as is the prediction for MC179 based on the present structural analysis. The connecting helices in MC179 extend out from the H1-H3 side of the bundle. In DBL1α, we predict that the connecting helices would also extend out from the H1-H3 side of the bundle. This is distinct from the H1-H2 side that interacts with the N-terminal bundle (“subdomain 2” [41]) (Figures 5A, 6). The overlay of the DBL domain and MC179 in Figure 5A also serves as a model for DBL1α, if one looks at the connecting helices of MC179 as modeling the predicted connecting helices of DBL1α. In addition, it is possible that the DBL1α and CIDR1α domains interact with each other in a manner resembling the MC179 dimer in the crystal.

Two additional points support the DBL1α-CIDR1α domain boundary as described here. First, Plasmodium proteins frequently have regions of low sequence complexity inserted in loops and between domains [47],[48]. In the PFL1960w 3D7 PfEMP1 sequence, there is an insertion of 110 glycine and serine residues after the multiple cysteines that mark the end of the DBL1α H3 helix and before the start of the CIDR1α domain. Binding studies with the CIDR1α domain from PFL1960w showed that it does bind CD36 [21], indicating that the insertion did not interfere with CD36 binding. This is consistent with the locations of the end of the DBL1α and the beginning of the CIDR1α domain as proposed here. Second, although MC179 is the minimal portion of the MC CIDR1α that binds CD36, two other recombinant CIDR1α domains bind CD36 only after the domains are lengthened in the N-terminal direction to be longer than MC179 [37]. The minimal lengthening needed to enable these two other CIDR1α domains to bind CD36 extended to just before the end of the DBL1α domains as predicted in this work. This is consistent with the boundary between the DBL1α and CIDR1α domains that does not include the DBL1α H3 helix in the CD36-binding CIDR1α domain.

### The DBLβ-C2 Domain

In addition to DBL and CIDR domains, a third type of domain, C2, is found in PfEMP1 molecules. There are nineteen C2 domains in the 3D7 genome and each is located C-terminal to a DBLβ domain. The A4var PfEMP1, containing a typical C2 domain, is known to bind ICAM-1, an endothelial protein that is a ligand of PfEMP1 [49]. When each of the A4var DBL and CIDR domains were expressed using domain boundaries based on sequence conservation, none of the expressed domains bound ICAM-1 [26]. The PfEMP1 domain that bound ICAM-1 was identified by combining DBL2β with the following C2 domain [50]. In subsequent studies to identify the minimal DBL2β-C2 domain that allowed ICAM-1 binding, the C-terminus of the C2 domain was progressively truncated [51]. ICAM-1 binding was lost when a truncation removed, as would be predicted from the current work, the third helix of the three-helix bundle at the C-terminus of the DBL2β-C2 domain. Work with a *P. falciparum* parasite that binds ICAM-1 also demonstrated that both DBL2β and C2 domains are required for ICAM-1 binding, as neither DBL2β or C2 expressed alone was able to bind ICAM-1 [52]. From these results, we suggest that the C2 domain completes the subdomain 3 of the DBLβ domain, by providing the connecting residues and the third helix of the three-helix bundle that has the cysteines that make disulfides to conserved cysteines in the H2 helix. That the C2 domain contains the third helix of the DBLβ subdomain 3 has been predicted recently from homology modeling [53].

### PfEMP1 Is a Polymer of Three-Helix Bundles

Sequences of PfEMP1 proteins vary enormously to evade the host immune response, while maintaining their binding to endothelial receptors. Conservation of binding to CD36 in the midst of tremendous variation in CIDR1α sequences implies an essential role for binding in parasite survival and in disease outcome for the host. Based on the MC179 structure and the DBL structures known at present, the highly conserved helices and disulfide bonds of the three-helix motif comprise the individual domains of the molecules in the PfEMP1 and EBA families and serve as the scaffolding on which sequence variation takes place. The two helical bundles in DBL domains and the single bundle in CIDR domains imply that a PfEMP1 molecule is not only modular in domain organization, but is a polymer of helical bundles, elaborated by connecting loops and helices. These loops and helices exhibit extraordinary sequence polymorphism and may be regions exposed to the bloodstream and its antibodies. Focusing on the binding of host ligands by PfEMP1 molecules will give insights into vaccine or drug strategies that will affect cytoadherence, help elucidate the ways that the parasite adjusts the functioning of the immune system, and should provide tools to lessen the impact of malaria in endemic areas of the world.

## Materials and Methods

### MC179 Protein Expression and Crystallization

MC179 (residues 576–754 of GenBank U27338) and MC167 (residues 576–742) expression plasmids were transformed into BL21 DE3-(RIL) cells, grown to an OD_600_ of ∼1, and induced with IPTG to produce insoluble protein (inclusion bodies). To initiate refolding of either MC167 or MC179, guanidine-solubilized inclusion bodies (30 mg) were then diluted to 45 ml with 3 M guanidine-HCl and pumped (0.05 ml/min) into 1 L of refolding buffer containing 400 mM arginine-HCl, 100 mM Tris-Cl, 5 mM cystamine-HCl, 2 mM DTT, and 2 mM NaEDTA. After 24 h, cystamine-HCl (50 mM) was added and allowed to react for 1 h to block free cysteines. The solution was then dialyzed against 10 L of water for 24 h and then against 10 L of 10 mM Tris-HCl pH 8.0 for 24 h. Protein was concentrated on CM52 cation exchange resin using 10 mM MES-NaOH at pH 6.0 and purified on a Superdex 75 size exclusion column. Protein fractions were concentrated to 6 mg/ml with CentriPrep concentrators. Using the hanging drop method, bipyramidal hexagonal MC167 or MC179 crystals grew in 27% polyethylene glycol (PEG) 400, 100 mM NaCl, 50 mM sodium citrate, pH 4.2, and diffracted to 2.7 Å using X-rays from a rotating anode generator. PEG400 (30% v/v) cryoprotected the frozen crystals. Seleno-methionine-containing inclusion bodies were produced in minimal medium (Athena Enzyme Systems, FL) using the BL21-(DE3)-X strain that is auxotrophic for methionine. Selenomet-MC167 or MC179 was refolded and purified similarly to native and the presence of selenium was verified by mass spectrometry. Selenomethionine crystals grew in 15% PEG400, 100 mM NaCl, and 50 mM sodium citrate, pH 4.2.

### X-Ray Data Collection and Structure Determination

Native, selenomethionine, and heavy atom derivative X-ray data were integrated and scaled with the XDS package [54] (Table 1). Seleno-methionine positions were found with SHELXD [55]. The native, selenium, ytterbium, and osmium datasets were used in SHARP [56] to find and refine additional heavy atom sites and to estimate protein phases for electron density maps. After density modification with RESOLVE [57], clear helical segments were observed in the electron density. Using the selenium positions as landmarks, the model was built, and then refined in the CNS package [58] to an R_work_ value of 0.25 and an R_free_ value of 0.29. No dependable model could be built for one N-terminal residue, loop residues 30–43, and C-terminal residues 168–179. Using model phases and highly redundant data collected with 1.54 Å X-rays, anomalous difference electron density confirmed the positions of each sulfur atom in the model. Models were superimposed with LSQMAN [59] and the SSM server [60]. Figures were produced with PyMOL software [61] and Jalview [62]. Surface area calculations were performed with the PISA server [63]. Coordinates and structure factors have been deposited with accession code 3C64 in the RCSB Protein Data Bank.

**Table 1:** X-ray data and structure refinement statistics.

	Se-Met	YbCl_3_	K_2_OsO_4_	Native
Space Group	P6_5_22	P6_5_22	P6_5_22	P6_5_22
Unit cell dimensions				
**a, b, c** (Å)	95.6, 95.6, 85.5	94.6, 94.6, 84.6	94.7, 94.7, 84.6	93.3, 93.3, 85.7
Wavelength (Å)	0.97925	1.38586	0.99998	1.5418
Resolution (Å)	38–2.8 (3.0–2.8)	37–2.4 (3.2–2.8)	37–2.8 (2.9–2.8)	41–2.4 (2.5–2.4)
*R*_sym_ (%)	22.5 (118)	5.5 (20.5)	6.4 (15.0)	12.6 (51.1)
*I*/σ*I*	16.9 (3.7)	21.6 (7.9)	40.1 (7.0)	18.8 (5.4)
Completeness (%)	99.5	99.8	99.4	99.9
Redundancy	28.9	6.3	21.8	18.2
**Refinement**				
Resolution (Å)				20–2.4
No. of reflections				9,044
*R*_work_/*R*_free_				0.25/0.29
No. of atoms				
Protein				1,216
Ligand/ion				14
Water				23
Average B-values (Å^2^)				
Protein				41.6
Ligand/ion				43.6
Water				42.3
R.M.S. deviations				
Bond lengths (Å)				0.009
Bond angles (°)				1.1
Ramachandran (%)				
Favored, allowed				93.6, 5.0
Generous, disallowed				0.0, 1.4

### CD36 Binding Assay

The binding of recombinant MC179 with a hexa-His tag at its C-terminus to human CD36, expressed on stably transfected Chinese hamster ovary cells (CHO-CD36), was assayed with a flow cytometry protocol similar to one described [64]. Briefly, cells were suspended at 10^6^ cells/ml in phosphate-buffered saline (PBS) containing 0.5% bovine serum albumin and 0.1% (w/v) sodium azide (FCA buffer). Cells (100 µl) in V-bottomed, 96-well plates were pelleted, then resuspended in 100 µl containing 100 ng/ml MC179 and incubated for 1 h. Following washing with PBS, an Alexa 488-labeled anti-pentahis monoclonal antibody (100 µl) (Novagen, San Diego, CA) or an anti-MC179 serum from an immunized *Aotus* monkey were added at a final concentration of 0.8 µg/ml and 1∶100, respectively. IgG1 isotype mAb (Becton Dickinson, San Jose, CA) and *Aotus* pre-immune serum were included as controls. After incubation for 30 min, cells were washed twice in PBS and *Aotus* antibodies were detected with Alexa 488-labeled goat anti-human IgG (Invitrogen, Carlsbad, CA) (100 µl) diluted 1∶250 in FCA buffer. The cells were then incubated for 30 min, washed, resuspended in 150 µl and analyzed by FACSort (Becton Dickinson). After staining with 0.5 µg/ml propidium iodide (PI), PI^+^ cells were excluded from the analysis. Anti-CD36 antibody clones used were 185-1G2 (Lab Vision, Fremont, CA), CLB-IVC7 (Sanquin, Amsterdam, Netherlands), and 8A6 [36] (gift from Dr. J. W. Barnwell, Center for Disease Control, Atlanta, GA). All the washing steps and incubations were performed at 4°C. Events (10,000) were acquired using CELLQuest software (version 3.3; Becton Dickinson) and the data from the live cell gate were analyzed by FlowJo software (version 6.4.1; Tree Star, San Carlos, CA).

## Supporting Information

Figure S1 The CIDR1α sequences from strain 3D7 aligned with the MC179 sequence. The alignment was produced with ClustalW and colored in clustalx colors in the alignment editor/viewer Jalview (http://www.jalview.org/). The MC179 helices are positioned according to the MC179 sequence (top line) and have colors and labels as described in the text. In the CIDR1α domains of 3D7, there are a few duplications, so that PFD1000c_CIDR1α is not in the alignment as it is identical to PFD0995C_CIDR1α and MAL13P1.1_CIDR1α is not present as it is identical to PFE0005w_CIDR1α.
(125 KB PDF)

Figure S2 All of the CIDR sequences from strain 3D7 aligned with the MC179 sequence. The alignment was produced with ClustalW and colored in clustalx colors in the alignment editor/viewer Jalview (http://www.jalview.org/). The MC179 helices are positioned according to the MC179 sequence (top line) and have colors and labels as described in the text.
(182 KB PDF)

Figure S3 The CIDR2β sequences from strain 3D7 aligned with the MC179 sequence. Note that the connecting helices region of CIDR2β is less variable in length than in CIDR1α sequences. Note that some CIDR2β sequences lack cysteines 45 and 49 (MC179 numbering), for example, PF11_0007. In most domains, these cysteines make conserved disulfides with Cys 159 and Cys 168 or the analogous residues. The alignment was produced with ClustalW and colored in clustalx colors in the alignment editor/viewer Jalview (http://www.jalview.org/). The MC179 helices are positioned according to the MC179 sequence (top line) and have colors and labels as described in the text. MAL8P1.207_CIDR2β is not in the alignment as it is identical to PFD1235w_CIDR2β.
(119 KB PDF)

Figure S4 Plot of the lengths of the connecting helices of DBL1α and CIDR1α domains. The *x*-axis lists the 47 PfEMP1 genes from 3D7 that contain DBL1α-CIDR1α pairs, or head structures. The *y*-axis plots the lengths of the connecting helices of DBL1α (red) and CIDR1α (blue). The line (red) is a best-fit line to the DBL1α data. The 47 data pairs have a Spearman's rank-order correlation coefficient between them of 0.58 with *p*<0.0001.
(98 KB PDF)
