# Supplementary figures and images for: The Cysteine-Rich Interdomain Region from the Highly Variable Plasmodium falciparum Erythrocyte Membrane Protein-1 Exhibits a Conserved Structure
Source: PLoS Pathog. 2008 Sep 5;4(9):e1000147. doi: 10.1371/journal.ppat.1000147 (PMC2518858; doi:10.1371/journal.ppat.1000147)

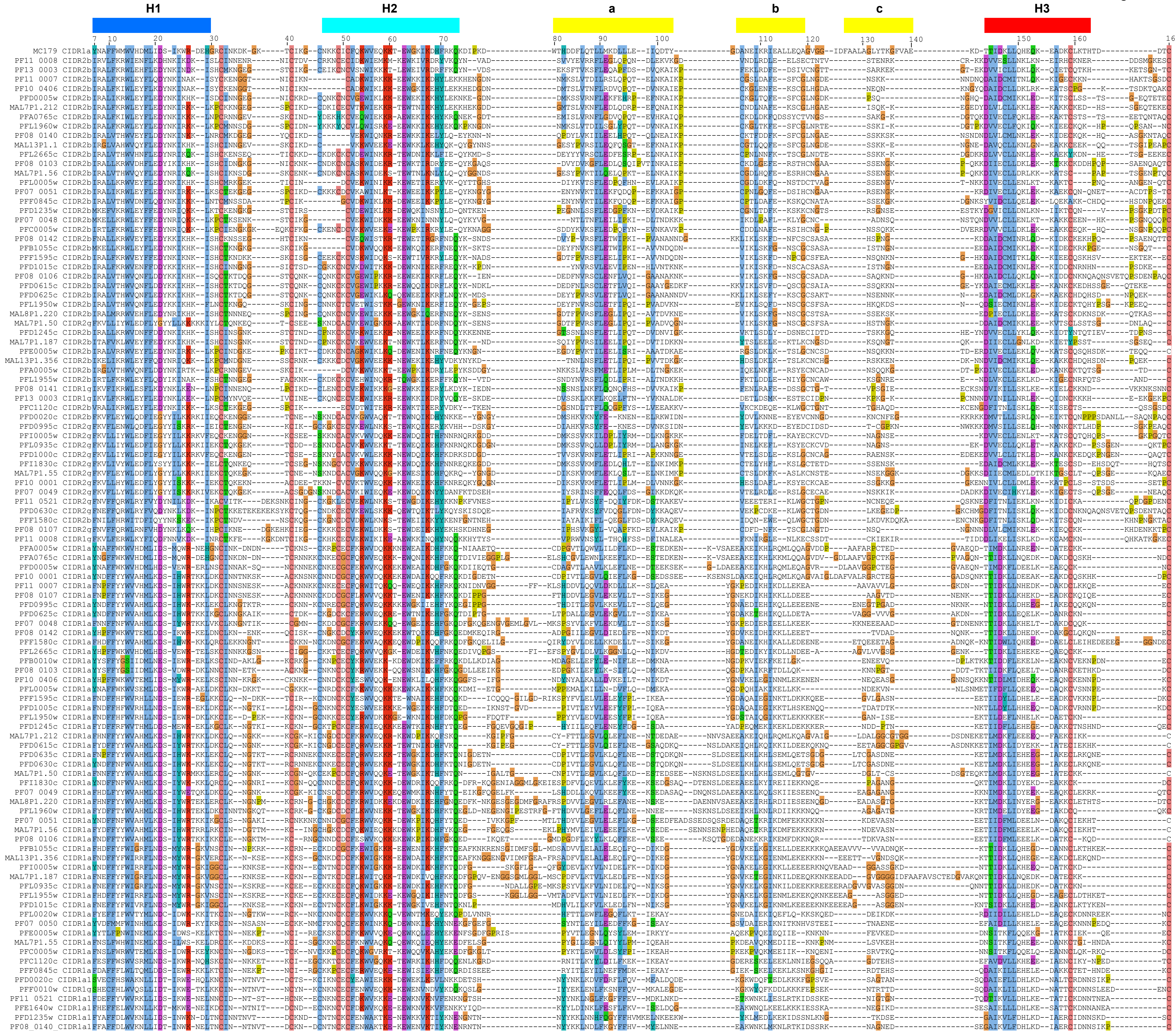

Supplement: Figure S2 — All of the CIDR sequences from strain 3D7 aligned with the MC179 sequence. The alignment was produced with ClustalW and colored in clustalx colors in the alignment editor/viewer Jalview (http://www.jalview.org/). The MC179 helices are positioned according to the MC179 sequence (top line) and have colors and labels as described in the text. (182 KB PDF) [file ppat.1000147.s002.pdf]

Figure S4

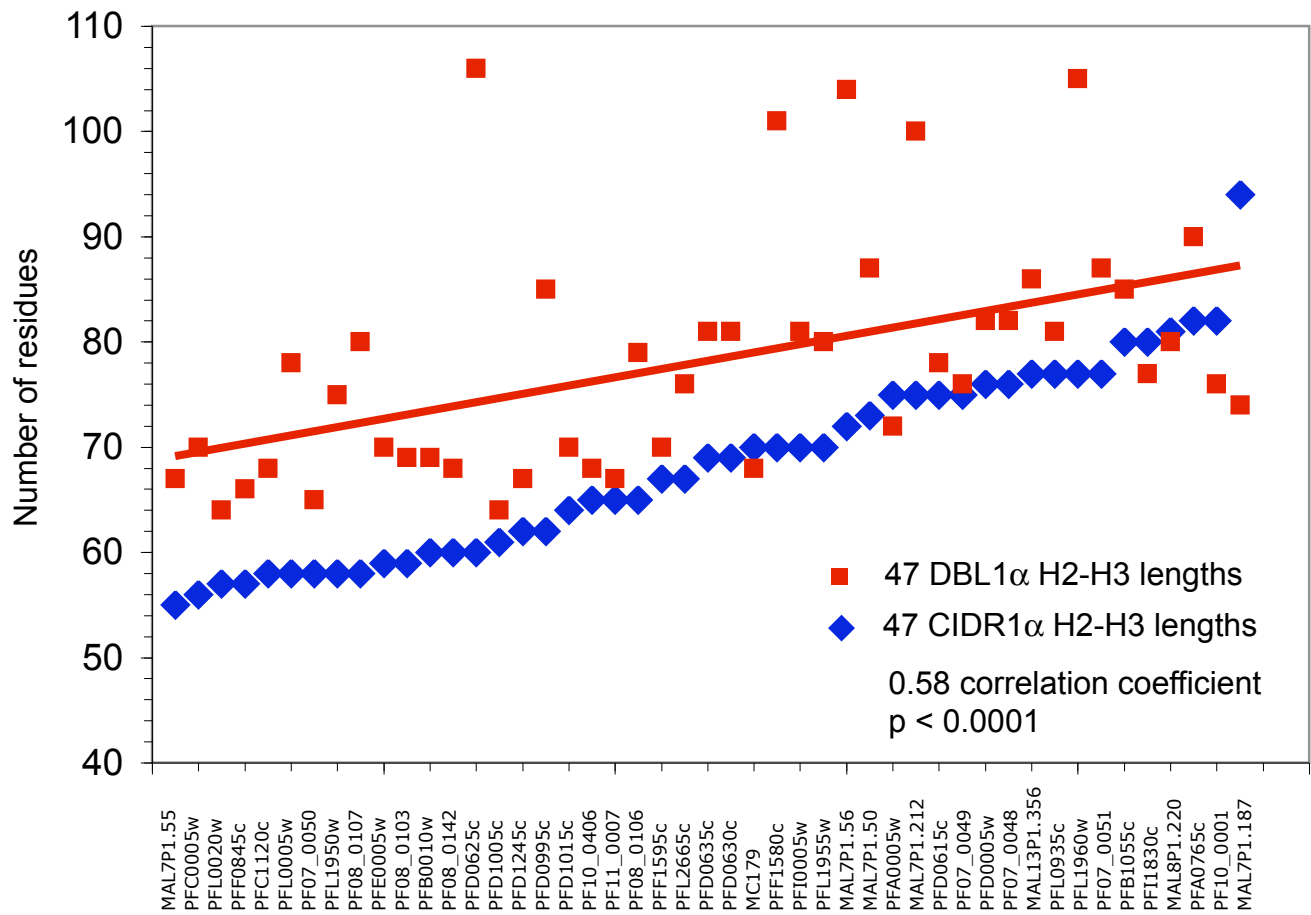

Supplement: Figure S4 — Plot of the lengths of the connecting helices of DBL1α and CIDR1α domains. The x-axis lists the 47 PfEMP1 genes from 3D7 that contain DBL1α-CIDR1α pairs, or head structures. The y-axis plots the lengths of the connecting helices of DBL1α (red) and CIDR1α (blue). The line (red) is a best-fit line to the DBL1α data. The 47 data pairs have a Spearman's rank-order correlation coefficient between them of 0.58 with p<0.0001. (98 KB PDF) [file ppat.1000147.s004.pdf]
