# Supplementary material for: The Cysteine-Rich Interdomain Region from the Highly Variable Plasmodium falciparum Erythrocyte Membrane Protein-1 Exhibits a Conserved Structure
Source: PLoS Pathog. 2008 Sep 5;4(9):e1000147. doi: 10.1371/journal.ppat.1000147 (PMC2518858; doi:10.1371/journal.ppat.1000147)
Supplement: Figure S3 — The CIDR2β sequences from strain 3D7 aligned with the MC179 sequence. Note that the connecting helices region of CIDR2β is less variable in length than in CIDR1α sequences. Note that some CIDR2β sequences lack cysteines 45 and 49 (MC179 numbering), for example, PF11_0007. In most domains, these cysteines make conserved disulfides with Cys 159 and Cys 168 or the analogous residues. The alignment was produced with ClustalW and colored in clustalx colors in the alignment editor/viewer Jalview (http://www.jalview.org/). The MC179 helices are positioned according to the MC179 sequence (top line) and have colors and labels as described in the text. MAL8P1.207_CIDR2β is not in the alignment as it is identical to PFD1235w_CIDR2β. (119 KB PDF) [file ppat.1000147.s003.pdf]

Figure S3

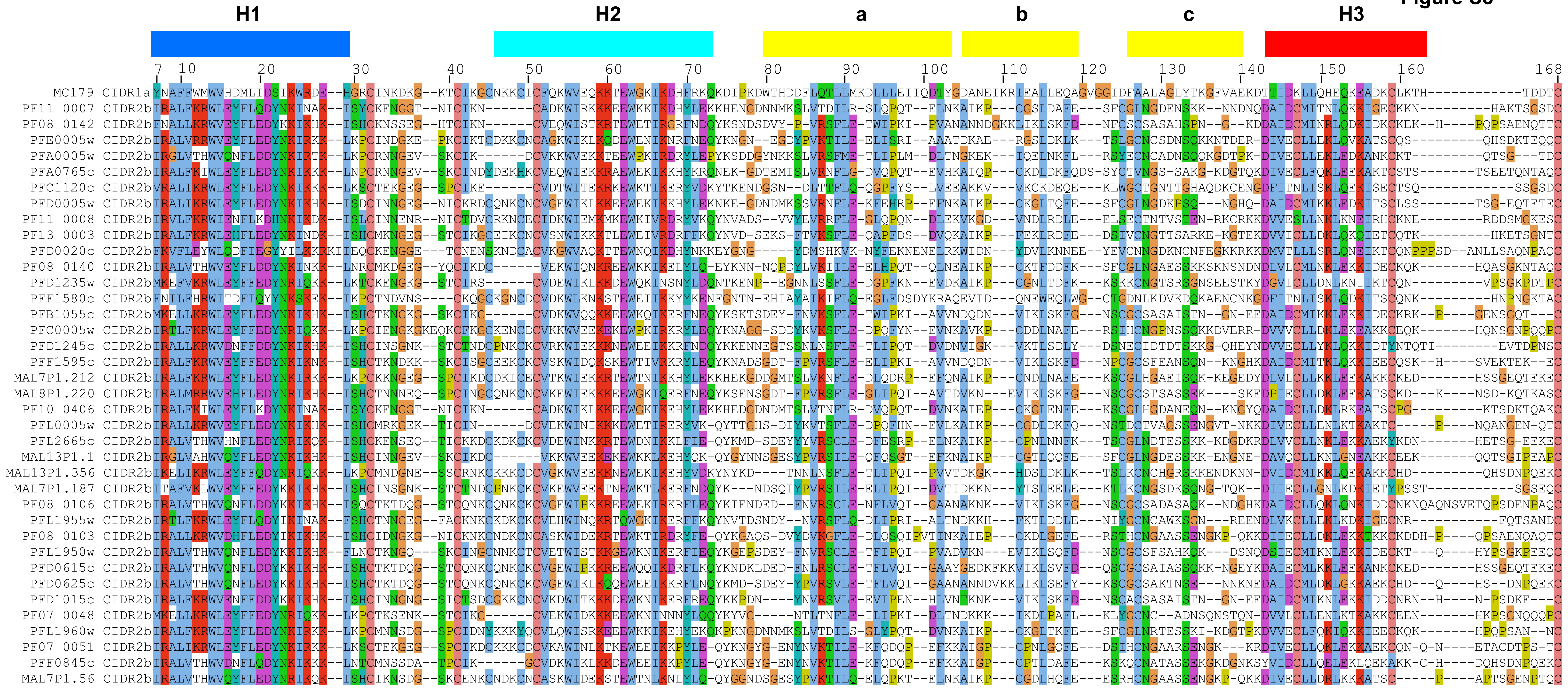
H1
H2
a
b
c
H3
7
10
20
30
40
50
60
70
80
90
100
110
120
130
140
150
160
168
MC179 CIDR1a
PF11_0007 CIDR2b
PF08_0142 CIDR2b
PFE0005w CIDR2b
PFA0005w CIDR2b
PFA0765c CIDR2b
PFC1120c CIDR2b
PFD0005w CIDR2b
PF11_0008 CIDR2b
PF13_0003 CIDR2b
PFD0020c CIDR2b
PF08_0140 CIDR2b
PFD1235w CIDR2b
PFF1580c CIDR2b
PFB1055c CIDR2b
PFC0005w CIDR2b
PFD1245c CIDR2b
PFF1595c CIDR2b
MAL7P1.212 CIDR2b
MAL8P1.220 CIDR2b
PF10_0406 CIDR2b
PFL0005w CIDR2b
PFL2665c CIDR2b
MAL13P1.1 CIDR2b
MAL13P1.356 CIDR2b
MAL7P1.187 CIDR2b
PF08_0106 CIDR2b
PFL1955w CIDR2b
PF08_0103 CIDR2b
PFL1950w CIDR2b
PFD0615c CIDR2b
PFD0625c CIDR2b
PFD1015c CIDR2b
PF07_0048 CIDR2b
PFL1960w CIDR2b
PF07_0051 CIDR2b
PFF0845c CIDR2b
MAL7P1.56_CIDR2b
