# Supplementary material for: The Cysteine-Rich Interdomain Region from the Highly Variable Plasmodium falciparum Erythrocyte Membrane Protein-1 Exhibits a Conserved Structure
Source: PLoS Pathog. 2008 Sep 5;4(9):e1000147. doi: 10.1371/journal.ppat.1000147 (PMC2518858; doi:10.1371/journal.ppat.1000147)
Supplement: Figure S1 — The CIDR1α sequences from strain 3D7 aligned with the MC179 sequence. The alignment was produced with ClustalW and colored in clustalx colors in the alignment editor/viewer Jalview (http://www.jalview.org/). The MC179 helices are positioned according to the MC179 sequence (top line) and have colors and labels as described in the text. In the CIDR1α domains of 3D7, there are a few duplications, so that PFD1000c_CIDR1α is not in the alignment as it is identical to PFD0995C_CIDR1α and MAL13P1.1_CIDR1α is not present as it is identical to PFE0005w_CIDR1α. (125 KB PDF) [file ppat.1000147.s001.pdf]

Figure S1

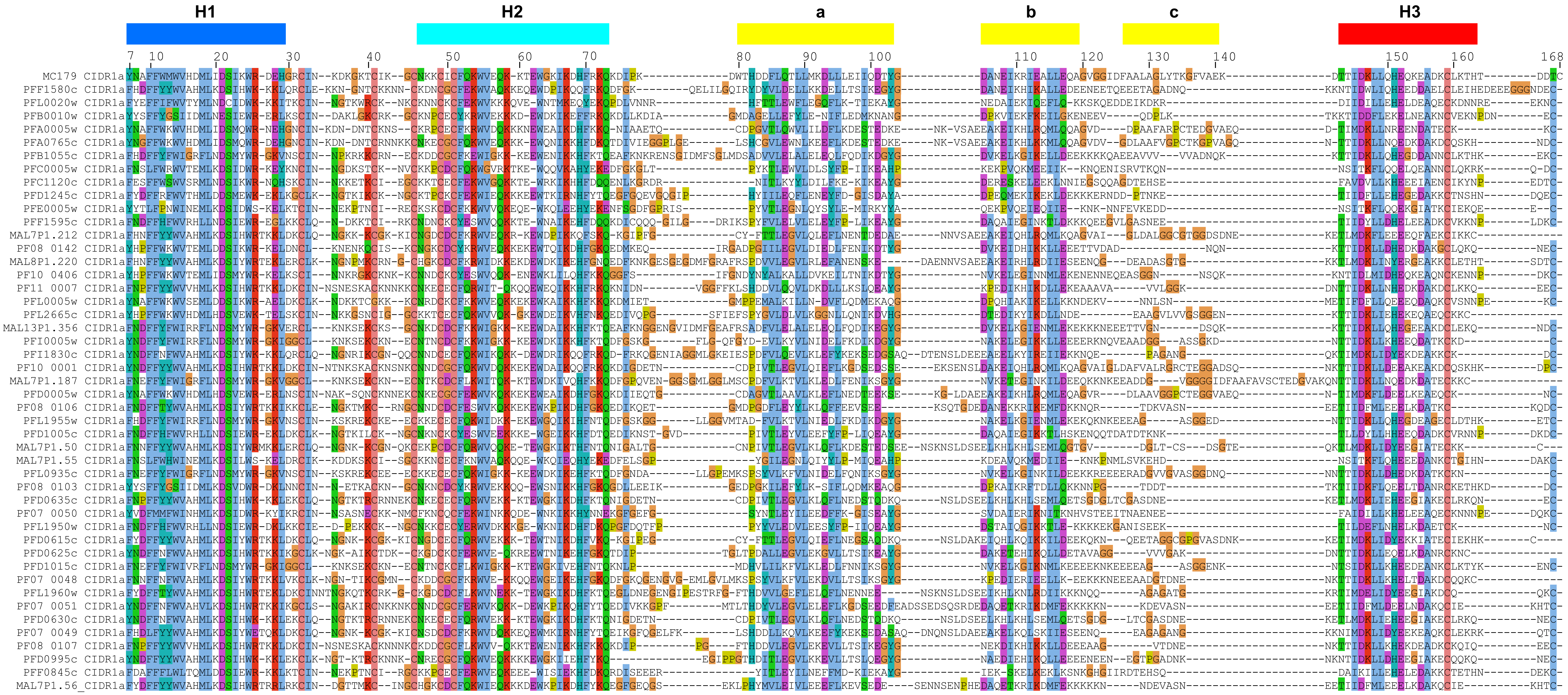
H1
H2
a
b
c
H3
7 10 20 30 40 50 60 70 80 90 100 110 120 130 140 150 160 168
MC179 CIDR1a
PFF1580c CIDR1a
PFL0020w CIDR1a
PFB0010w CIDR1a
PFA0005w CIDR1a
PFA0765c CIDR1a
PFB1055c CIDR1a
PFC0005w CIDR1a
PFC1120c CIDR1a
PFD1245c CIDR1a
PFE0005w CIDR1a
PFF1595c CIDR1a
MAL7P1.212 CIDR1a
PF08_0142 CIDR1a
MAL8P1.220 CIDR1a
PF10_0406 CIDR1a
PF11_0007 CIDR1a
PFL0005w CIDR1a
PFL2665c CIDR1a
MAL13P1.356 CIDR1a
PFI0005w CIDR1a
PFI1830c CIDR1a
PF10_0001 CIDR1a
MAL7P1.187 CIDR1a
PFD0005w CIDR1a
PF08_0106 CIDR1a
PFL1955w CIDR1a
PFD1005c CIDR1a
MAL7P1.50 CIDR1a
MAL7P1.55 CIDR1a
PFL0935c CIDR1a
PF08_0103 CIDR1a
PFD0635c CIDR1a
PF07_0050 CIDR1a
PFL1950w CIDR1a
PFD0615c CIDR1a
PFD0625c CIDR1a
PFD1015c CIDR1a
PF07_0048 CIDR1a
PFL1960w CIDR1a
PF07_0051 CIDR1a
PFD0630c CIDR1a
PF07_0049 CIDR1a
PF08_0107 CIDR1a
PFD0995c CIDR1a
PFF0845c CIDR1a
MAL7P1.56_CIDR1a
